# A deadly equation: The global toll of US TB funding cuts

**DOI:** 10.1371/journal.pgph.0004899

**Published:** 2025-09-10

**Authors:** Sandip Mandal, Sreenivas Nair, Suvanand Sahu, Lucica Ditiu, Carel Pretorius

**Affiliations:** 1 Center for Modeling and Analysis, Avenir Health, Glastonbury, Connecticut, United States of America; 2 Stop TB Partnership, Geneva, Switzerland; Human Sciences Research Council, SOUTH AFRICA

## Abstract

The recent withdrawal of U.S. financial support threatens essential TB service delivery, including diagnostics, treatment, TB-HIV co-infection interventions and research initiatives critical to eradicating TB. This study analyses the dependency of and potential impact of funding cuts to 26 high-burden TB countries (HBCs). We modelled three recovery scenarios: (1) minimal impact (services recover within three months), (2) moderate impact (recovery within one year), and (3) worst-case scenario (long-term service reduction). Extrapolations were made for all 26 HBCs based on representative countries from each dependency category. Across all 26 HBCs, additional TB cases between 2025 and 2030 are estimated at 0.63 million (CI 0.45–0.81) (minimal impact), 1.66 million (CI 1.2–2.1) (moderate impact), and 10.67 million (CI 7.85–13.19) (worst-case). Corresponding TB deaths are projected to increase by 99,900 (CI 65,200–130,000), 268,600 (CI 185,800–337,900), and 2,243,700 (CI 1,570,800–2,807,300), respectively. The loss of U.S. funding endangers global TB control efforts, jeopardizing progress towards End TB and SDG targets, and potentially puts millions of lives at risk. While some nations may adapt, short-term disruptions will severely impact vulnerable populations. Urgent alternative funding is needed to sustain critical TB prevention and treatment efforts.

## Introduction

The recent U.S. foreign aid cuts have significantly impacted global health programs including HIV, malaria, and tuberculosis (TB). This has led to the closure of numerous essential aid programs worldwide [1,2]. This includes the suspension of HIV treatment services, malaria prevention initiatives, and TB care programs, leaving millions without access to critical healthcare services with expected negative impact on disease burden [3–5]. The abrupt funding discontinuation by the US Government (USG) has impacted TB care and prevention across high TB burden countries [6]. In 2024, U.S. contributions accounted for over 55% of the total external funding, highlighting their critical role in the global TB response (Section A and Fig A in S1 Appendix) [7].

USAID Programs funded by USAID’s TB initiative now face severe disruptions in key areas, including detection and treatment of TB and drug-resistant TB; expanded coverage of interventions for TB-HIV co-infection; prevention and treatment of TB infection; improvements in the TB service delivery platforms;— all elements essential to the Global Plan to End TB (2023–2030) [8]. The 2030 projection horizon of this analysis was selected to align with these targets, which outlines strategic priorities and funding needs to accelerate progress towards ending the TB epidemic by 2030. Additionally, critical TB research, essential for developing new tools and achieving the 2030 SDG targets, is under threat. USAID has been a key funder for TB drug and diagnostics development, operational research, and vaccine innovation [7]. The aid discontinuation jeopardizes these advancements, potentially stalling progress in the fight against TB.

In the face of these challenges, using mathematical modelling, we estimated the potential impact of U.S. TB funding cuts on 26 countries with high TB burden and who are reliant on foreign aid for TB service delivery. In the following section we describe the basic model framework, the different data sources involved and model scenarios. We present results for projected epidemics on service disruptions under different scenarios for the speed of recovery in financial support. Finally, we discuss implications of this work, the limitations of the model and global relevance.

## Methods

### Analysis of data on reliance

The degree of dependency on US Government (USG) funding for tuberculosis (TB) programs varies widely across countries. Our analysis focused on 26 high-burden countries (HBC), major recipients of the US international aid, using publicly available expenditure data reported annually by countries to WHO, to estimate an approximate distribution for the proportion of total expenditure that derives directly from USG [9] Funding contributions were relatively stable in the period from 2021 to 2023. Accordingly, we used three-year average expenditure data from 2021-2023 where available (15 countries), and 2023 data if data from 2021 or 2022 were missing (11 countries).

We categorized these countries into three levels of dependency. Category boundaries were chosen such that an approximately equal number of countries fall in each of the three categories:

- Low Dependency (0%–22% of NTP expenditure funded by USG): Eight out of 26 HBCs have diversified funding sources with minimal reliance on US aid, making them less susceptible to disruptions in TB services.
- Moderate Dependency (23%–37% of NTP expenditure funded by USG): Ten countries out of 26 HBCs face moderate risk, where reductions in USG funding could strain budgets, affecting service delivery and supply chains.
- High Dependency (>37% of NTP expenditure funded by USG): Eight countries out of 26 HBCs are highly reliant on USG funding and are extremely vulnerable to program disruptions, including shortages of diagnostic tools, medications, and healthcare personnel (Table A in S1 Appendix). A freeze and discontinuation in aid poses a severe threat to the continuity of TB services in these nations.

It should be noted that these 26 high-burden countries account for approximately 80% of the Global TB burden and 90% of the TB burden within the Global Fund’s TB portfolio (and, consequently, among countries dependent on foreign aid for delivering TB services).

### Model

We investigated the potential impact of funding cuts on TB incidence and mortality by modelling service disruptions using a deterministic, compartmental model of TB transmission under a Bayesian framework (Section B and Fig B in S1 Appendix) [10]. The model was first calibrated using country-specific data (Table B and Table C in S1 Appendix). To assess the impact of service disruption, we applied a method similar to the one used to evaluate the effects of service disruption on TB during the COVID-19 pandemic [11]. This approach was later adopted by WHO for burden estimation from 2020 onward [12]. We assumed that the proportion of NTP expenditure funded by USG will translate proportionately to impact on parameters that influence detection and treatment outcomes. A 10% funding reduction, for instance, would decrease key probabilities in the TB detection cascade by 10% in 2025.

In our analysis, and likely in practice, there is no immediate way to overcome the funding gap and service delivery disruption, sooner than three months after the 90 days evaluation period of the USAID funding freeze. Even in situations where countries can fund the gap themselves, it would take time to restructure procurement and logistics systems. Therefore, we explored three recovery scenarios to estimate the potential impact of a funding freeze and discontinuation.

a. Minimum Impact Scenario (S1): The USAID freeze and funding cut disrupts TB services for 90 days. The funding gap is subsequently met, and the country recovers service coverage to baseline levels within the next three months.
b. Moderate Impact Scenario (S2): The USAID freeze and funding cut disrupts TB services for 90 days. The funding gap is subsequently met, and it takes one year for the country to recover service coverage to baseline levels.
c. Worst Possible Scenario (S3): The USAID freeze and funding cut disrupts TB services for 90 days, after which the funding gap is not met, and service coverage remains at the lower level reached during the 90-day period.

To better illustrate the potential epidemiological effects of a funding freeze and discontinuation, we chose one country from each dependency category–Country 1: A country with high TB burden and low dependency on USG funding; Country 2: A country with high TB burden with moderate dependency on USG funding; and Country 3: A country with high TB burden and high dependency on USG funding. We calibrated the respective country models with epidemiological data (Fig C in S1 Appendix) for these countries and simulated TB incidence and mortality under the three disruption scenarios (Table B, and Fig D in S1 Appendix).

To estimate the impact for the remaining 23 high-burden countries, we used a simple extrapolation method, as also applied under previous analysis for the potential impact of disruptions during COVID-19 [11]. Baseline trends for new TB cases and TB deaths were projected using a cubic-spline method, and each were assigned to the aforementioned-dependency category (low, medium and high) [13]. We then applied the relative impact estimates from the representative country of each category to the baseline trend of the country being extrapolated.

Our best estimate is derived from the impact mapping of each country, based on funding contributions reported to WHO. The range of estimates is generated by randomly varying the USAID dependency level assigned to each country–classified as low, medium, or high USAID dependency. This interval is then rescaled so that the median impact level from these variations aligns with the best estimate. This approach serves as a practical method to account for uncertainty in mapping countries to a representative (or directly modelled) country and the uncertainty in our assumption that a proportional reduction in funding contributions directly corresponds to a similar reduction in essential TB services. A sensitivity analysis is presented around this assumption.

## Results

In 2023, total funding for tuberculosis (TB) care and prevention reached US$ 5.7 billion, with 21% (US$ 1.2 billion) coming from external sources. This external funding played a crucial role in sustaining TB programs worldwide. In 2024, USAID committed US$ 406 million (Fig A in S1 Appendix) [14,15], while the Global Fund (GF) allocated US$ 800 million, of which approximately one-third (~US$ 267million) originated from the U.S. Government.

Across all scenarios, service disruptions lead to delays in diagnosis and treatment initiation, resulting in substantial increases in TB incidence and mortality (Fig D in S1 Appendix). The impact of funding cuts under three different scenarios across three country categories, as shown in Table 1. The increase in TB cases and deaths varies significantly depending on the country category. For instance, in Country 1, the minimum increase in cumulative cases is 0.5% (CI: 0.3%–0.7%), with cumulative deaths rising by 0.8% (CI: 0.6%–1.0%) between 2025–2030. In contrast, under the worst-case scenario in Country 3, cumulative cases could surge by 36% (CI: 25%–47%), while deaths could increase by 68% (CI: 45%–86%) between this period.

**Table 1:** Percentage increase in cumulative TB cases and deaths across three country categories under three impact scenarios.

	Percentage increase of cumulative TB cases between 2025–2030			Percentage increase of cumulative TB deaths between 2025–2030		
	Minimal impact scenario	Moderate impact scenario	Worst impact scenario	Minimal impact scenario	Moderate impact scenario	Worst impact scenario
Country 1	0.5 (CI 0.3–0.7)	1.2(CI 0.9–1.6)	6.8(CI 4.6–9.1)	0.8 (CI 0.6–1.0)	2.0 (CI 1.5–2.5)	13.2 (CI 9.6–17.0)
Country 2	1.2 (CI 0.8–1.6)	3.1(CI 2.2–4.3)	19(CI 12.7–27.4)	1.9 (CI 1.4–2.4)	4.9 (CI 3.5–6.4)	37 (CI 25–52)
Country 3	2.1 (CI 1.5–2.5)	5.4 (CI 4.0–6.5)	36 (CI 25 –47)	2.9 (CI 2.2–3.5)	7.8 (CI 5.9–9.2)	68 (CI 45–86)

**Fig 1:**
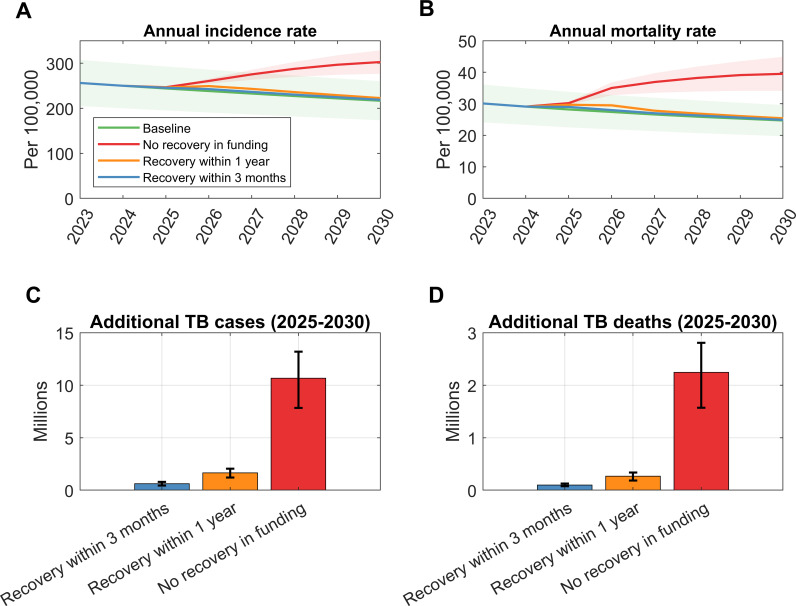
Impact of a US Aid freeze and cut on tuberculosis outcomes across 26 HBCs from 2025–2030. (A) Annual TB incidence per 100,000 population under three funding scenarios: a 90-day freeze with a subsequent 3-month recovery, a one-year recovery, and a long-term disruption (no recovery). (B) Annual TB mortality rate corresponding to the same three recovery scenarios. (C) and (D) shows additional cases and deaths during 2025 to 2030 under the three recovery scenarios.

Across the 26 high-burden countries, Scenarios S1-S3 are estimated to result in 634,700 (CI 447,700–806,600), 1,660,000 (CI 1,210,400–2,060,000), and 10,676,400 (CI 7,848,800–13,190,000) additional TB cases, respectively, from 2025 to 2030 (Fig 1A and 1C, also see Table 2). Correspondingly, TB deaths are projected to increase by 99,900 (CI 65,200–130,000), 268,600 (CI 185,800–337,900), and 2,243,700 (CI 1,570,800–2,807,300) across these scenarios during the same period (Fig 1B and 1D, also see Table 2).

**Table 2:** Impact of short-, medium- and long-term disruption resulting from US funding freeze for TB.

	Uninterrupted scenario (S0)	Minimum impact scenario (S2)	Moderate impact scenario (S2)	Worst impact scenario (S3)
	Baseline	90-day disruption, 90- day recovery	90-day disruption, 1 year recovery	Long-term disruption
**New TB cases**				
Total for 2025–2030	49,656,500	50,291,200	51,316,500	60,333,000
Additional new TB cases relative to baseline		634,700 [447,700-806,000]	1,660,000 [1,210,400-2,060,000]	10,676,400 [7,848,800-13,190,000]
Annual increase		105,800 [74,600-134,300]	276,700 [201,700-343,300]	1,779,400 [1,308,100-2,198,300]
**TB Deaths**				
Total for 2025–2030	5,710,800	5,810,700	5,979,400	7,954,500
Additional TB deaths relative to baseline		99,900 [65,200-130,000]	268,600 [185,800-337,900]	2,243,700 [1,570,800-2,807,300]
Annual increase		16,700 [10,900-21,700]	44,800 [31,000-56,300]	374,000 [261,800-467,900]

We conducted a sensitivity analysis for which we assume that only 50% of the proportion of NTP expenditure funded by USG would proportionately influence the parameters affecting detection and treatment outcomes (Table D in S1 Appendix). In the worst-case scenario, under this assumption, the estimated additional cases are 4,385,500 (CI: 3,224,000–5,418,000) and additional deaths are 884,000 (CI: 618,900–1,106,100) (Table D in S1 Appendix).

## Discussion

With TB services having recovered to pre-COVID-19 levels, a new, potentially greater threat to TB SDG targets has emerged. The freeze and subsequent, sharp cut in U.S. funding has caused uncertainty within the global TB community, and threatens the ambitious goals outlined in the End TB Strategy and the operational framework set out in the TB Global Plan 2023–2030. The Global Plan highlights the critical role of new tools, including the development and rollout of a large-scale vaccine for TB, in achieving the End-TB targets [16]. Given the key role of the US as a key funder for TB research, withdrawal of USG support threatens the development of such critical new tools, further delaying progress and–without adequate mitigation–making End TB targets unattainable. Hard-to-reach, high-risk and vulnerable populations, previously connected to national TB programs through civil society organizations (CSOs), now face complete isolation as U.S.-funded initiatives shut down [17].

Pre-existing funding gaps have now widened, worsening resource shortages needed to combat TB. Our results suggest that under the worst-case scenario, over 2 million lives could be at risk in the next 6 years (Fig 1). This scenario is extreme, but now credible: with additional, high-income countries also cutting back on international aid [18,19], new options are needed for mitigating the shortfall in USG funding. Low-burden countries can also be impacted, either directly or indirectly, by funding cuts. A WHO–Lancet Microbe commentary highlights that the withdrawal of USAID TB funding would reduce case finding and treatment efforts, thereby increasing the risk of transmission, even in countries currently classified as low-burden, such as the United States [20].

A key limitation is the estimation of the U.S. contribution to total TB expenditure and the assumption that its withdrawal leads to a proportional reduction in essential TB services. We estimated the proportion of TB expenditure funded by the U.S. using published data from the WHO’s datasets [9]. However, the reliability of these 2023 proportions to approximate proportions in future years remains uncertain, as does the extent to which they directly support essential TB services, as assumed. To mitigate this, in estimating impact across all HBCs, we incorporated a lower-bound estimate by varying the impact category assigned to each country. However, even this conservative bound under a long-term disruption scenario remains highly concerning. In the coming months, it will be critical to monitor TB services in the affected countries, for direct evidence on the extent of service disruptions.

Our sensitivity analysis on the relationship between funding and service delivery, which assumed that only 50% of the USG-funded proportion of NTP expenditure would affect programmatic outcomes, revealed substantially lower impacts on TB burden compared to the main scenarios. Under this assumption, the projected additional TB cases and deaths in the worst-case scenario dropped by approximately 59% and 61%, respectively, compared to the original estimates. This suggests that the estimated health impact is sensitive to the assumed magnitude of USG funding influence on detection and treatment parameters. A recent modelling study in low- and middle-income countries estimated a much smaller impact of funding cuts, primarily because it assumed that the cuts would affect only treatment initiation [21] In contrast, the current study assumes that funding reductions also impact overall case-finding activities, leading to a larger estimated effect.

While our base-case assumptions represent a plausible scenario based on historical programmatic influence, these findings underscore the importance of further empirical country-engaged work to better quantify the relationship between donor funding levels and specific programmatic outcomes. Nonetheless, even under conservative assumptions, the projected increase in TB burden remains considerable, reinforcing the critical role of sustained financial support for national TB programs.

While our extrapolation method enables broad comparisons across countries, it does not explicitly account for contextual country differences such as health system infrastructure, political stability, or the degree of donor diversification. These factors may influence the actual impact of funding disruptions and are important to consider when interpreting our results. We therefore emphasise the need for country-level engagement to enhance the relevance and utility of our modelling for national decision-making.

While our model implicitly includes drug-resistant TB (DR-TB) within the overall TB burden, we did not separately analyse the effects of funding reductions on DR-TB diagnosis, treatment initiation, and outcomes. Given the substantially higher costs, longer treatment durations, and lower success rates associated with DR-TB, disruptions to programmatic support could disproportionately affect these patients. This represents an important limitation of our analysis, and future modelling efforts should seek to explicitly capture the impact of funding shocks on the DR-TB care cascade.

The impact of disruptions and cuts to HIV service delivery under the PEPFAR program is expected to lead to additional HIV infections and to HIV disease progression, which in turn will lead to an increase in HIV-TB burden in many countries with high joint burden of HIV and TB. While our models are calibrated to total TB burden and implicitly includes HIV and TB co-morbidity, we did not account for the expected increases in HIV burden and for the additional TB cases and deaths that will result. This is another source of under estimation in our impact results, especially in a worst-case scenario for HIV and TB programs.

No coordinated modelling project has yet assessed the impact of funding cuts on TB, unlike for the impact on HIV due to funding cuts, where such cuts are estimated to cause over 3 million additional deaths [22]. A rough estimate suggests 0.9 million of these could be HIV-TB deaths, well above the implicit estimate from our results, as detailed in Section E in S1 Appendix.

The WHO database for TB expenditure, which we used to estimate USAID’s contribution, includes Global Fund (GF) sources (to which USAID contributes one-third) but captures only about 50% of the non-GF congressional appropriation for TB. We did not incorporate the remaining 50% in our analysis due to a lack of detailed information on its allocation across countries and its contribution to essential TB services. As a result, any potential overestimation that arises from our assumption that a proportional funding reduction leads to an equivalent reduction in essential TB services is partially offset by this omission.

TB death estimates here therefore appear reasonable, though based on simplified assumptions and limited to three directly modelled countries. Country-specific differences and alternative funding sources were not fully accounted for, highlighting the need for deeper, country-engaged analysis.

In optimistic scenarios where countries recover within three months or one year, they may shift to a low-dependency category and undergo service delivery improvements- a process not accounted for in our future and medium impact estimates. This leads to a broader point, which is not explored in this short- and medium-term impact analysis. The abrupt discontinuation of aid, no matter if or how it may be eventually restored, may force many countries to rethink their dependency not just on US but on all foreign aid, and invest in new strategic planning for key health areas, not just TB. And several options are available to this end, including increased domestic financing (which is expected to compel service integration across health areas) and refinancing their program through concessional loans from development banks and possibly regional financing platforms.

While the medium-term impact we studied here is alarming, the long-term picture may be more optimistic with countries becoming more self-reliant and more resilient to disruptions. Moreover, the recent funding cuts are expected to catalyse the integration of health programs, and the end of the siloed approach to health care programs that still characterize healthcare service delivery in many parts of the world.

Our analysis shows that across 26 high-burden countries which account for 80% of the global TB burden and 90% among recipients of US funding for TB, termination of US funding could result in an estimated 10.6 million additional TB cases and 2.2 million additional TB deaths during the period 2025–2030. The global TB response now stands at a crossroads: one path leading to continued reliance of foreign aid, while the other moves toward self-reliance and sustainability.

## Supporting information

S1 Appendix Model description and technical details. (DOCX)
